# Supplementation with the Extract of Schisandrae Fructus Pulp, Seed, or Their Combination Influences the Metabolism of Lipids and Glucose in Mice Fed with Normal and Hypercholesterolemic Diet

**DOI:** 10.1155/2014/472638

**Published:** 2014-04-30

**Authors:** Xiao-Yan Wang, Zhi-Ling Yu, Si-Yuan Pan, Yi Zhang, Nan Sun, Pei-Li Zhu, Zhan-Hong Jia, Shu-Feng Zhou, Kam-Ming Ko

**Affiliations:** ^1^Department of Pharmacology, School of Chinese Materia Medica, Beijing University of Chinese Medicine, Beijing 100102, China; ^2^School of Chinese Medicine, Hong Kong Baptist University, Hong Kong; ^3^Department of Pharmaceutical Sciences, College of Pharmacy, University of South Florida, FL 33612, USA; ^4^Division of Life Science, Hong Kong University of Science & Technology, Hong Kong

## Abstract

Schisandrae Fructus (SF), which possesses five tastes: sweet (fruit skin), sour (pulp), bitter/pungent (seed core), and saltiness (all parts), can produce a wide spectrum of biological activities in the body. Here, we investigated the effects of the ethanolic extract of SF pulp, seed, or their combination (namely, EtSF-P, EtSF-S, or EtSF-P/S, resp.; collectively called EtSF) on the metabolism of lipids and glucose in normal diet- (ND-) and hypercholesterolemic diet- (HCLD-) fed mice. Supplementation with EtSF significantly reduced hepatic triglyceride and cholesterol levels by 18–47% in both ND- and HCLD-fed mice. EtSF supplementation reduced serum triglyceride levels (approximately 29%), whereas EtSF-P and EtSF-S/P elevated serum cholesterol (up to 26 and 44%, resp.) in HCLD-fed mice. Treatment with EtSF decreased hepatic glucose levels (by 9–44%) in both ND- and HCLD-fed mice. Supplementation with EtSF-S or EtSF-S/P (at 1 and 3%) increased biliary or fecal TC contents in HCLD-fed mice. However, supplementation with EtSF-S/P at 9% reduced biliary TC levels in HCLD-fed mice. EtSF-P or EtSF-S/P supplementation reduced serum alanine aminotransferase activity in HCLD-fed mice. The findings suggested that supplementation with EtSF lowered lipid and glucose accumulation in the liver and increased fecal cholesterol contents in mice. Dietary supplementation with EtSF-P or EtSF-S/P attenuated liver damage in HCLD-fed mice.

## 1. Introduction

Hyperlipidemia (HLD) refers to increased levels of lipids in the blood, including cholesterol and triglyceride. It is well known that HLD, the leading cause of death and disability over the world, significantly increases the risk of cardiovascular diseases, nonalcoholic fatty liver disease (NAFLD), metabolic syndrome, stroke, or cerebrovascular accident [1–3]. NAFLD, which is the most common liver disease in western countries and with a clinical manifestation of steatosis and nonalcoholic steatohepatitis, is also recognized as a cause of cryptogenic cirrhosis and hepatocellular carcinoma [4]. A recent study has shown that the incidence of highly differentiated colorectal adenocarcinoma in NAFLD group is significantly higher than that in the control group [5]. In addition, NAFLD is associated with increased risk of hypertension [6], higher incidence of type 2 diabetes [7], and high levels of serum uric acid [8].

Since the incidences of HLD and NAFLD are increasing rapidly, it is of therapeutic interest to search for effective agents that can lower lipid contents in the blood and liver [9]. Although much effort has been put to develop drugs used for the prevention and treatment of HLD-related diseases, effective drugs for treating HLD, especially NAFLD, are yet to be discovered. In addition, synthetic lipid-lowering drugs have many potential adverse/side effects, such as muscle tenderness [10, 11], renal failure [12], and others, including headache, bowel upset, nausea, sleep disturbances, and hepatomegaly [13, 14]. In recent years, people prefer to use natural remedy such as dietary supplement/functional food for the prevention and treatment of NAFLD and/or lipid disorders [15, 16].

Schisandrae Fructus (SF, Wu-Wei-Zi in Chinese) has been used for thousands of years as a “superior” drug in the practice of Chinese medicine in China. Previous studies have shown that SF and its related chemical components possess a wide spectrum of biological activities such as antioxidation, antitumor [17, 18], hepatoprotection against chemically and virally induced hepatic injury [19], antifatigue, immunostimulation, and antiaging [20]. A recent study has demonstrated that SF extract can prevent ethanol-induced fatty liver, possibly through activation of AMPK (AMP kinase) and peroxisome proliferator-activated receptor *α* (PPAR*α*) signaling pathway [21]. Gomisin N, a diastereomer of schisandrin B (Sch B, a major active ingredient of SF), inhibited DNA damage checkpoint signaling by stereospecifically interacting with ataxia telangiectasia and Rad-3-related (ATR) protein kinase [22]. Our previous studies have shown that SF extracts [23, 24], Sch B [25], bicycol [26], and bifendate [27] can reduce hepatic triglyceride (TG) and total cholesterol (TC) levels in hypercholesterolemic (HCL) mice. In the present study, we endeavored to compare the effects of ethanol extracts of SF pulp, seed, or a mixture of pulp/seed on serum and hepatic lipid/glucose (GLU) levels, as well as liver function in mice under the normal and HCL conditions. Fenofibrate (FF) was used as a positive control for comparison.

## 2. Materials and Methods

### 2.1. Herbal Material and Extraction Procedure

SF, which is the fruit of* Schisandra chinensis* (Turcz.) Baillon (Bei-Wu-Wei-Zi in Chinese), was purchased from the Anguo Chinese herbs market in Hebei province, China, and authenticated by Professor Chun-Sheng Liu at the Beijing University of Chinese Medicine. The fruit pulp and seed were manually separated and then dried at room temperature. The weight of pulp and seed was 63 and 37% of total weight, respectively. For the preparation of SF extracts, SF pulp, seed, or both were crushed into small pieces using an industrial grinder and extracted twice (first, 1.5 h; second, 2 h) with 5 volumes of 80% (v/v, in H_2_O) ethanol under reflux after soaking for half an hour. The pooled extract was filtered by filter paper and concentrated by rotary evaporation to obtain the SF seed ethanolic extract (EtSF-S), SF pulp ethanolic extract (EtSF-P), and their combination extracts (EtSF-S/P). The extracts were stored at 4°C until use.

### 2.2. Chemicals and Regents

Cholesterol (certificate number 20120614) and bile salt (certificate number 20121210) were purchased from Sinopharm Chemical Reagent Co., Ltd. (Beijing, China). FF (certificate number 18246) was obtained from Beijing Yongkang Medical Ltd. (Beijing, China). Assay kits for TC, TG, and GLU were bought from Zhongsheng Beikong Biotechnology and Science Inc. (Beijing, China). Assay kits for high-density lipoprotein cholesterol (HDL), low-density lipoprotein cholesterol (LDL), and alanine aminotransferase (ALT) were purchased from Beijing Leadman Biochemistry Co., Ltd. (Beijing, China).

### 2.3. Animal and Treatment

All experimental procedures were approved by the University Committee on Research Practice at Beijing University of Chinese Medicine. Male ICR mice (grade II, certificate number SCXK (jing) 2012-0001), weighing 18–20 g, were supplied by Vital River Lab Animal Co. Ltd. (Beijing, China). All animals were maintained on a 12 h (light on 700–1900 h) light-dark cycle at 20–21°C, with a relative humidity of 50–55%. They were allowed for free access to water and food.

### 2.4. Experimental Design

#### 2.4.1. Design 1

In this study, the effects of dietary supplementation with SF extracts or FF on lipids, GLU, and liver were investigated in normal mice. Animals were divided into four groups of 10 animals in each: (1) mice fed with normal diet (ND); (2) and (3) mice fed with diet supplemented with 1% and 9% SF extracts (w/w), respectively; (4) mice fed with diet supplemented with 0.1% FF. After 10 days, mice were sacrificed under light ether anesthesia. Blood, collected from the orbital vein, and liver tissue samples were obtained and subjected to biochemical analysis.

#### 2.4.2. Design 2

**Figure 1:**
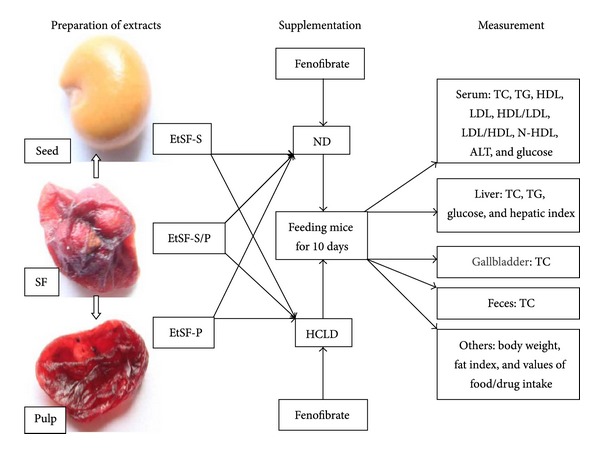
The design of the current study. SF: Schisandra Fructus; EtSF-S: ethanolic extract of SF seed; EtSF-P: ethanolic extract of SF pulp; EtSF-S/P: ethanolic extract of SF seed/pulp; TC: total cholesterol; TG: triglyceride; LDL: low-density lipoprotein; HDL: high-density lipoprotein; N-HDL: non-HDL; ALT: alanine aminotransferase; HCLD: hypercholesterolemic diet; ND: normal diet.

This study was designed to investigate the effects of SF extracts on serum and hepatic parameters in mice fed with HCL diet (HCLD) containing cholesterol/bill salt (1/0.3%, w/w). Mice were randomly divided into six groups (10 in each group): (1) mice fed with ND; (2) mice fed with HCLD; (3), (4), and (5) mice fed with HCLD supplemented with 1, 3, and 9% EtSF-S, EtSF-P, or EtSF-S/P, respectively; (6) mice fed with HCLD supplemented with 0.1% FF. Ten days later, animals were sacrificed and blood/liver tissue samples were collected for biochemical analysis.  Figure 1 shows the design of the present study.

### 2.5. Serum and Hepatic Biochemical Analysis

Serum samples were prepared by centrifuging the clotted blood for 8 min at 2000 ×g and stored at −20°C until used for biochemical analysis. Liver tissue samples were homogenized in 9 volumes of 0.9% (w/v) NaCl solution by two 10 s bursts of a tissue disintegrator at 13,500 rpm and then centrifuged at 2000 ×g for 15 min to obtain the supernatants. Ten *μ*L of serum and 40 *μ*L of the hepatic supernatant were used to determine the TG and TC levels with GPO-PAP and COD-PAP methods, respectively. Ten *μ*L serum and 5 *μ*L hepatic supernatant were used to determine the GLU levels with GOD-POD method. Serum HDL and LDL levels, as well as ALT activity, were determined using automatic biochemistry analyzer (Beckman Coulter Synchron CX4 PRO.Brea, CA, USA).

### 2.6. Measurement of Biliary and Fecal TC Contents

Mouse gallbladder was removed from the liver and soaked in 1 mL TC reagent for 16 h. Then TC concentrations (*μ*mol/gallbladder) were measured using the method described above. For the determination of TC contents in feces, mouse feces were collected and dried at room temperature. Dried feces (approximately 30 mg) were extracted with 0.5 mL chloroform-methanol (1 : 1, v/v) mixture for 12 h and then centrifuged at 2000 ×g for 5 min to obtain the supernatants. Thirty *μ*L fecal supernatants were used to measure the TC levels (*μ*mol/g feces) using assay kit.

### 2.7. Measurement of Hepatic Index

Body and liver weights were measured. Hepatic index was estimated from the ratio of total liver weight to body weight (liver weight/body weight × 100).

### 2.8. Statistical Analysis

Values given are means ± SEM. Data were analyzed by one-way ANOVA using SPSS statistical analysis program and then differences among means were analyzed by Dunnett's multiple comparisons test or post hoc analysis. *P* < 0.05 was considered significant.

## 3. Results

### 3.1. Effects of EtSF Supplementation on Serum Lipid Profiles

As shown in Table 1, daily supplementation with EtSF (i.e., EtSF-S, EtSF-P, and EtSF-S/P) did not affect serum TC, TG, and HDL levels in mice fed with ND. However, both EtSF-P and EtSF-S/P supplementation markedly increased serum HDL and LDL levels (up to 15–47% and 14–73%, resp.) in ND- and HCLD-fed mice. All 3 tested EtSF extracts decreased serum TG levels (up to 25%) in HCLD-fed mice, but EtSF-P and EtSF-S/P markedly increased serum TC levels (approximately 26 and 44%, resp.) in mice fed with HCLD. Feeding mice with HCLD markedly increased serum TC, LDL, and N-HDL levels, as well as LDL/HDL ratio. HCLD decreased serum TG level (up to 60%) and HDL/LDL ratio, when compared with ND-fed mice. EtSF-S supplementation did not affect serum HDL/LDL and LDL/HDL ratios, but decreased N-HDL level in ND-fed mice. EtSF-P and EtSF-S/P supplementation decreased serum HDL/LDL ratio and increased LDL/HDL ratio and N-HDL levels in both ND-fed and HCBD-fed mice. FF supplementation reduced serum TC (32%), TG (52%), HDL (34%), or HDL/LDL ratio but increased LDL/HDL ratio, in normal mice. Serum TC, TG, LDL, and N-HDL levels were reduced by 29, 38, 66, and 69%, respectively, in mice fed with FF-supplemented HCLD diet, when compared with those fed with HCLD only. Moreover, FF elevated serum HDL/LDL ratio but decreased LDL/HDL ratio in HCLD-fed mice.

### 3.2. Effects of EtSF Supplementation on Hepatic Lipid/Glucose Levels

**Figure 2:**
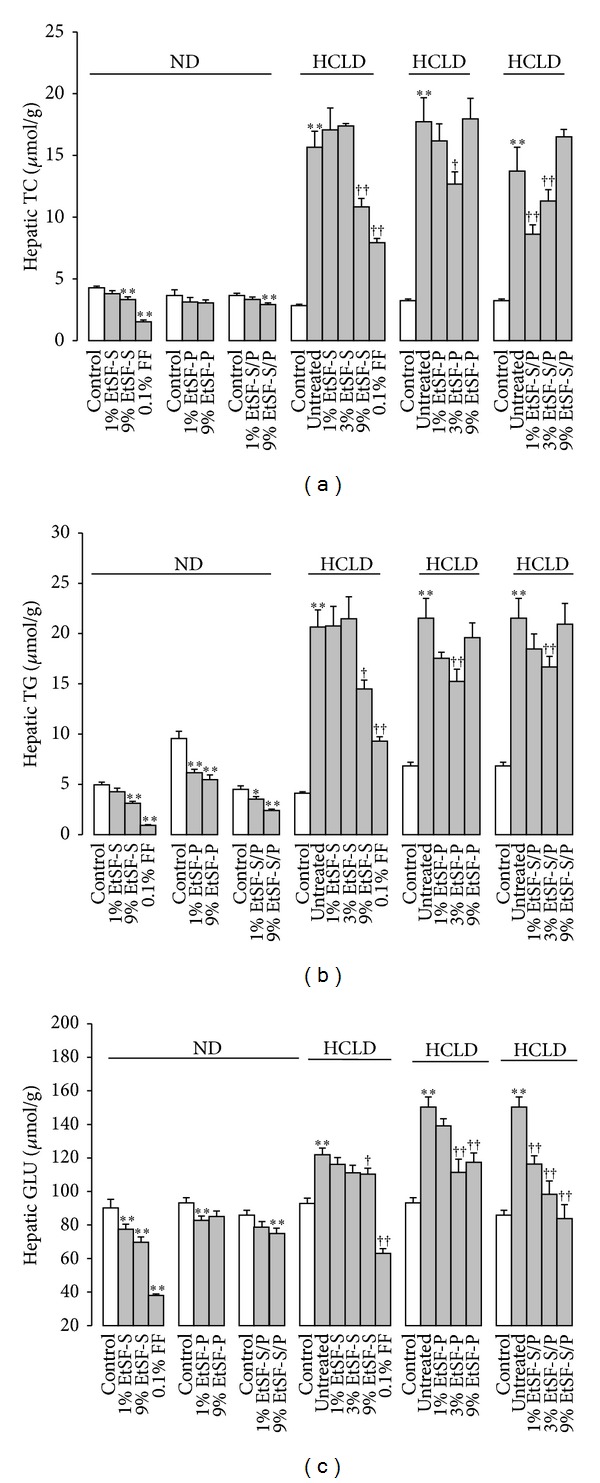
Effects of EtSF supplementation on hepatic lipid/glucose contents in normal and HCL mice. Experimental details were described in Table 1. Mice were fed with ND and HCLD without or with EtSF or FF supplementation, as indicated in the figure. Ten days later, hepatic TC (a), TG (b), and glucose (c) contents were measured. Values given are the means ± SEM, with *n* = 10. *P < 0.05, **P < 0.01 versus mice fed with ND and ^†^ *P* < 0.05, ^††^ *P* < 0.01 versus mice fed with HCLD alone. Statistically significant differences were determined using a one-way ANOVA followed by Dunnett's multiple comparisons test or post hoc analysis.

Supplementation with EtSF-S, EtSF-P, or EtSF-S/P decreased hepatic TC and TG contents (up to 47%) in ND-fed mice. Feeding mice with HCLD markedly increased hepatic TC and TG contents (up to 447 and 402%, resp.), when compared with those of mice fed with ND. Supplementation with EtSF-S, EtSF-P, or EtSF-S/P reduced the hepatic TC and TG contents by 18–37% and 23–30%, respectively, in HCL mice. FF supplementation lowered hepatic TC/TG contents by 64/49 and 81/55% in both normal and HCL mice, respectively (Figures 2(a) and 2(b)). Dietary supplementation with 3 tested SF extracts and FF reduced hepatic GLU contents by 10/44% and 58/44% in ND-/HCLD-fed mice, respectively (Figure 2(c)).

### 3.3. Effects of EtSF Supplementation on Biliary and Fecal Cholesterol Contents

**Figure 3:**
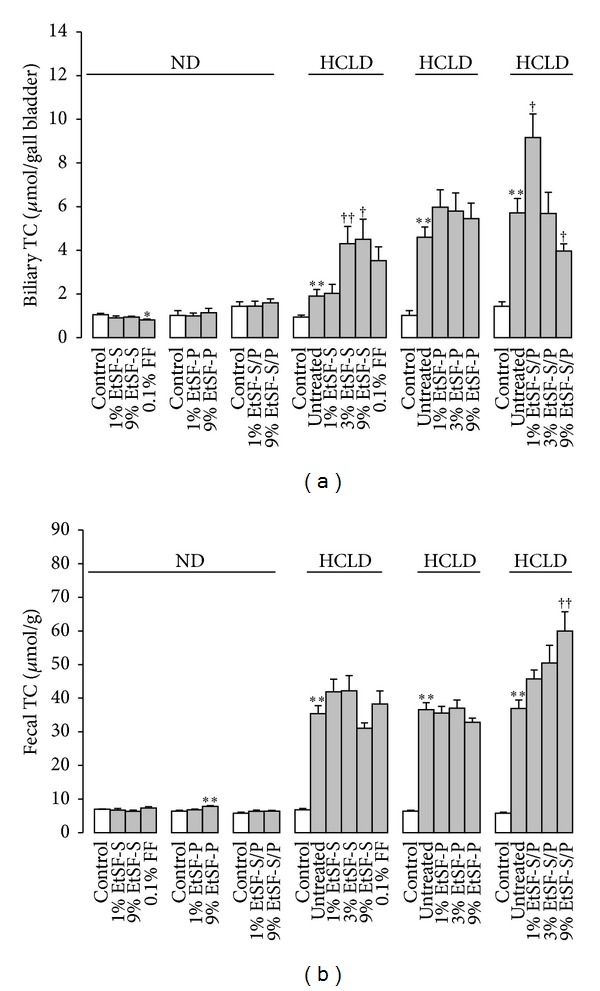
Effects of EtSF supplementation on biliary and fecal cholesterol contents in normal and HCL mice. Experimental details were described in Table 1. Mice were fed with ND and HCLD without or with EtSF or FF supplementation, as indicated in the figure. Ten days later, biliary (a) and fecal (b) TC contents were measured. Values given are the means ± SEM, with *n* = 10. *P < 0.05, **P < 0.01 versus mice fed with ND and ^†^ *P* < 0.05, ^††^ *P* < 0.01 versus mice fed with HCLD alone. Statistically significant differences were determined using a one-way ANOVA followed by Dunnett's multiple comparisons test or post hoc analysis.

EtSF-S and 1% EtSF-S/P, but not EtSF-P, supplementation increased biliary TC concentrations in HCLD-fed mice (up to 136 and 60%, resp.). However, supplementation with 9% EtSF-S/P reduced biliary TC by 30% in HCL mice (Figure 3(a)). Feeding mice with 9% EtSF-P and EtSF-S/P elevated the fecal cholesterol excretion (by 21 and 62%, resp.) (Figure 3(b)).

### 3.4. Effects of EtSF Supplementation on Hepatic Index and Function

**Figure 4:**
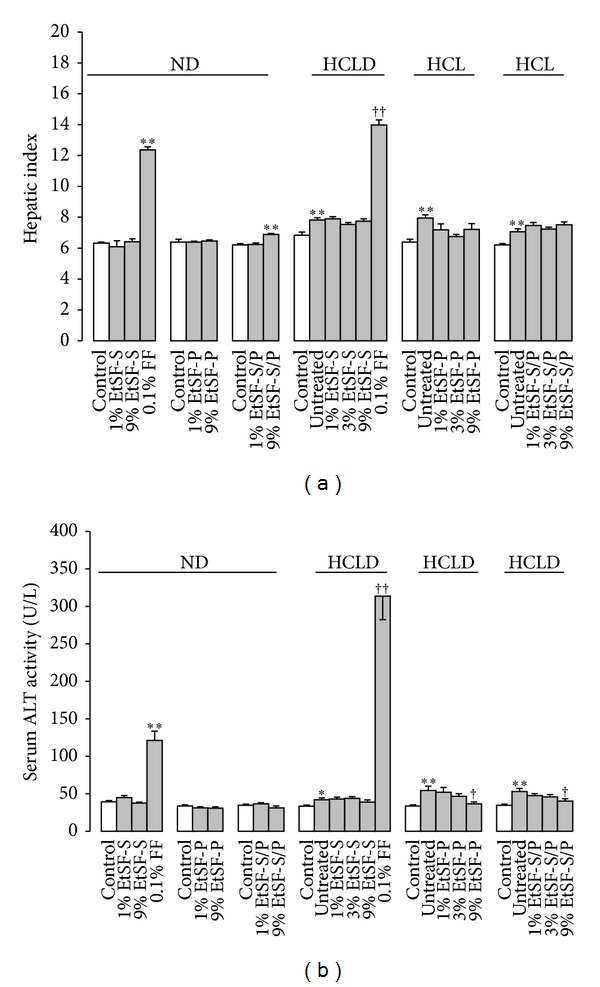
Effects of EtSF supplementation on hepatic index and function in normal and HCL mice. Experimental details were described in Table 1. Mice were fed with ND and HCLD without or with EtSF or FF supplementation, as indicated in the figure. Ten days later, hepatic index (a) and serum alanine aminotransferase (ALT) activity (b) were measured. Values given are the means ± SEM, with *n* = 10. *P < 0.05, **P < 0.01 versus mice fed with ND and ^†^ *P* < 0.05, ^††^ *P* < 0.01 versus mice fed with HCLD alone. Statistically significant differences were determined using a one-way ANOVA followed by Dunnett's multiple comparisons test or post hoc analysis.

Feeding mice with EtSF-S/P or HCLD increased hepatic index by 10 or 18%, respectively, when compared with control ND group. FF increased hepatic index by 95 and 79%, respectively, in normal and HCL mice, respectively (Figure 4(a)). EtSF did not alter the serum ALT activity in normal mice, but EtSF-P and EtSF-S/P lowered the ALT activity (33 and 24% decrease, resp.) in HCL mice. FF supplementation significantly elevated serum ALT activity by 209 and 650%, respectively, in ND- and HCLD-fed mice (Figure 4(b)).

### 3.5. Effects of EtSF Supplementation on Body Weight and Food/Drug Intake

EtSF-P supplementation decreased the body weight (up to 6%; *P* < 0.05) in ND-, but not HCLD-, fed mice. However, no detectable changes in body weight between EtSF-S/EtSF-S/P supplemented and unsupplemented mice fed with ND and HCLD were observed. In addition, weight loss was observed in FF-supplemented mice with ND (by 8%) and HCLD (by 16%). Daily intake of EtSF-S, EtSF-P, or EtSF-S/P was estimated to be 1.47–1.68 g/kg (based on crude herb equivalent) at 1% supplementation, 4.23–5.47 g/kg at 3% supplementation, and 12.72–15.43 g/kg, at 9% supplementation. The human equivalent dose of 1% EtSF is estimated to be 0.15–0.17 g crude herb/kg. The daily intake of FF was estimated to be 0.15 and 0.13 g/kg in normal and HCL mice, respectively (Table 2).

## 4. Discussion

While genetic inheritance may contribute to the development of HLD and its related NAFLD in some patients, the main pathological causes are related to the lack of exercise and diet with high levels of saturated fats and carbohydrates [3]. In the present study, mice fed with a diet containing cholesterol and bile salt for 10 days exhibited elevations in serum TC, LDL levels, and ALT activity, as well as hepatic TC, TG, and GLU levels, which were associated with hepatomegaly and liver injury. Biliary and fecal TC concentrations were also increased in mice fed with HCLD. It was observed that feeding mice with HCLD for ten days was able to increase serum LDL level but causes no detectable change in serum HDL. The findings indicated that HLD and/or NAFLD in humans were successfully mimicked by a mouse model of feeding HCLD. Nevertheless, the low levels of serum TG, as observed in the mouse model, may be related to the short period (10 days) of modeling, HCLD composition, and/or interspecies differences [28, 29]. In clinical situation, however, serum TG level is often not a concomitant parameter in HCL; it may increase or remain unchanged but never be lower than the normal range.

Given that N-HDL, HDL/LDL ratio, and LDL/HDL ratio are clinical parameters for assessing the risk of cardiovascular diseases, NAFLD, and metabolic syndrome in humans [30–32], these parameters were also adopted for evaluating the effectiveness of the tested EtSF extracts in mice fed with HCLD. Feeding mice with HCLD increased serum LDL/HDL ratio and N-HDL levels but decreased the HDL/LDL ratio, which are consistent with the clinical manifestation of HLD [33–35]. FF supplementation increased HDL/LDL ratio and decreased N-HDL levels and LDL/HDL ratio in HCL mice. On the other hand, the supplementation with EtSF-P and EtSF-S/P decreased HDL/LDL ratio and elevated N-HDL levels and LDL/HDL ratio. While EtSF-P and EtSF-S/P lowered serum TG and enhanced serum HDL, both EtSF extracts increased serum TC and N-HDL levels and LDL/HDL ratio, as well as decreased HDL/LDL ratio. These findings suggest that EtSF-P and EtSF-S/P (but not EtSF-S) supplementation may lead to further worsening of lipid parameters in mice under HCL condition. However, it has been reported that the baseline levels of plasma TC, HDL, LDL, and TG in mice were marginally higher than the reference ranges prior to the experiment and 2 weeks of EtSF supplementation did not cause any significant changes in lipid parameters [36].

It is well established that lipid metabolism is closely related to GLU metabolism in the body. The relevant metabolic disorders constitute the pathological basis of hyperlipidemia, metabolic syndrome, type 2 diabetes, fatty liver disease, and obesity [37]. In the present study, supplementation with EtSF-S, EtSF-P, or EtSF-S/P did not change serum TC and TG levels but altered hepatic lipid contents and GLU levels in HCL mice. While EtSF-P and EtSF-S/P supplementation increased hepatic TC contents in HCLD-fed mice, EtSF-S lowered the hepatic TC content. Serum levels of HDL and LDL (often referred to as “good” cholesterol and “bad” cholesterol, resp.) [38] were increased in mice fed with HCLD or ND supplemented with EtSF-P or EtSF-S/P. The elevation of serum HDL and LDL levels by EtSF extracts, in particular the EtSF-S/P, might result from a metabolic response to hypercholesterolemia, wherein the increased cholesterol content necessitates higher levels of LDL and HDL for transportation in the blood. Supplementation with EtSF was found to markedly decrease hepatic TC, TG, and GLU contents in both ND- and HCLD-fed mice. EtSF-S/P supplementation at 1% increased biliary TC level; however, the supplementation at 9% reduced biliary TC level and increased fecal TC excretion. While EtSF-S attenuated serum TG levels, both EtSF-P and EtSF-S/P did not cause any changes in normal and HCL mice. Taken together, the results suggest that SF can influence the lipid and GLU metabolism in mice in a complex manner, especially under the HCL condition.

A previous study in our laboratory has shown that Sch B lowered fat accumulation in L-02 cells incubated with free fat acid via inhibition of adipose differentiation-related protein (ADRP) and sterol regulatory element-binding protein (SREBP-1) expression [39]. It is known that ADRP is closely associated with intracellular lipid droplets and upregulated in hepatic steatosis [40]. SREBP-1 is the most important transcription factor regulating* de novo* lipogenesis in the liver and induces insulin resistance [41]. Kwon et al. [42] reported that SF lignans could improve insulin sensitivity via the PPAR-*γ* pathways. Therefore, it is possible that the EtSF extracts tested in the present study may protect against NAFLD and decrease hepatic GLU contents through a similar action mechanism. Hyperlipidemia is commonly associated with insulin resistance, which may result in hyperinsulinemia and hyperglycemia. However, in present study, feeding mice with HCLD did not increase serum GLU levels (data not shown). Instead, HCLD elevated hepatic GLU, which may be related to the stimulation of hepatic gluconeogenesis, an indicative of insulin resistance in extrahepatic tissues. In addition, based on the reduction of hepatic TC content and elevation of serum TC/LDL levels by EtSF extracts in HCLD-fed mice, it is possible that EtSF extracts, particularly the EtSF-S/P, can stimulate the release of TC from the liver and thereby ameliorate hepatic steatosis.

The observations of increased serum ALT activity, enlarged liver size, and increased lipid accumulation in the HCLD-fed mice suggest the presence of liver damage, which may result from the accumulation of lipids in hepatic tissue [43] and/or activation of signaling pathways in hepatocytes that stimulate the production of proinflammatory mediators [44]. Supplementation with EtSF-P and EtSF-S/P protected against liver damage in HCL mice, as evidenced by the decrease in serum ALT activity. It is believed that the dibenzocyclooctadiene-type lignans such as schisandrin A and Sch B are the active components of SF in protecting against liver injury [45]. As to why the lignan-enriched EtSF-S was unable to protect against liver damage in HCLD-fed mice remains to be investigated,

FF, the fibrates class of lipid-lowering drugs, is commonly used in the treatment of HLD as a PPAR*α* agonist for reducing cardiovascular risks and treating NAFLD/nonalcoholic steatohepatitis [46, 47], as well as improving the GLU tolerance and lowering adiposity [48]. Significant lowering of serum and hepatic lipid/GLU levels, as well as body weight and fat mass, was observed following FF supplementation (data not shown). As about one-fourth to one-third of blood cholesterol is carried by HDL, hence, low serum HDL levels caused by FF might result from the drug-induced hypocholesterolemia in normal mice. Although fibrate treatment improved liver function in patients with metabolic syndrome in clinic situation [49] and ameliorated concanavalin A-induced hepatitis in rats [50], FF can cause acute cholestatic hepatitis in patients [51–53]. In the present study, the daily supplementation with FF (130–150 mg/kg; about 30-fold higher than the human dose) induced hepatomegaly and increased ALT levels in normal and HCL mice. FF-induced elevation in serum ALT activity might be partly due to the increased expression of hepatic transaminase gene [54].

Herbal drugs, which contain a mixture of chemical components, can produce a wide spectrum of biological actions. According to the theory of Chinese medicine, SF possesses five tastes (Wu Wei in Chinese)—sweet (fruit skin), sour (pulp), bitter/pungent (seed core), and saltiness (all parts). SF pulp, which mainly contains polysaccharides/sugars and organic acids [55, 56] as well as dibenzocyclooctadiene lignans such as schisandrin A, B, and C and gomisin A and N, is responsible for producing most of the pharmacological activities. Although lignans are most abundantly found in SF seeds [57], EtSF-S is not the most biologically active among the 3 tested SF fractions, as observed in the present study. In addition, the pharmacological actions produced by EtSF do not always display a dose-response relationship. For instance, EtSF-S/P reduced hepatic GLU level in a dose-dependent manner, but it did not lower hepatic TC at the highest tested dose (i.e., 9%). While three doses (1, 3, and 9%) were tested in HCL mice, two doses were adopted in normal mice to examine the possible toxicity of EtSF in mice. Although the daily doses of 9% EtSF could reach about 15 g/kg/day for 10 days, they did not affect the behaviors in mice (data not shown).

In conclusion, results obtained from the present study showed that the supplementation with EtSF produced a significant influence on lipid/GLU metabolism in ND- and HCLD-fed mice, especially in HCL mice. Dietary supplementation with EtSF-P or EtSF-S/P elevated serum lipid levels, except for that of serum TG levels which was lowered, in HCL mice. Dietary supplementation with EtSF-S, EtSF-P, or EtSF-S/P reduced hepatic lipid and GLU concentrations in both normal and HCL mice. EtSF-S/P, but not EtSF-S and EtSF-P, supplementation increased fecal cholesterol excretion in HCLD-fed mice. EtSF-P and EtSF-S/P attenuated the HCLD-induced hepatotoxicity. Supplementation with FF decreased serum and hepatic lipid and GLU levels, as well as increased serum ALT activity and liver weight in mice fed with ND and/or HCLD (see the summary of results in Table 3). The ensemble of results indicates a differential effect between SF seed and pulp on lipid and GLU metabolism, particularly in HCL mice. Supplementation with EtSF might ameliorate the lipid accumulation in liver cells and thus protect against liver injury in HCL mice.

## Figures and Tables

**Table 1:** Effects of EtSF supplementation on serum lipid profiles in normal and HCL mice.

Groups	Drug (%)	TC (mmol/L)	TG (mmol/L)	HDL (mmol/L)	LDL (mmol/L)	HDL/LDL	LDL/HDL	N-HDL (mmol/L)
ND-fed mice								
ND	—	3.39 ± 0.10	1.24 ± 0.06	3.57 ± 0.12	0.44 ± 0.02	8.18 ± 0.35	0.13 ± 0.01	0.65 ± 0.02
ND/EtSF-S	1	3.37 ± 0.14	1.29 ± 0.08	3.66 ± 0.19	0.45 ± 0.02	8.27 ± 0.57	0.13 ± 0.01	0.53 ± 0.05*
	9	3.25 ± 0.12	1.05 ± 0.15	3.58 ± 0.14	0.45 ± 0.03	8.07 ± 0.43	0.13 ± 0.01	0.46 ± 0.05**
ND/FF	0.1	2.32 ± 0.10**	0.60 ± 0.03**	2.36 ± 0.14**	0.40 ± 0.03	6.12 ± 0.38**	0.17 ± 0.01**	0.68 ± 0.03
ND	—	4.23 ± 0.25	1.81 ± 0.05	3.64 ± 0.18	0.47 ± 0.03	7.90 ± 0.38	0.13 ± 0.01	0.71 ± 0.12
ND/EtSF-P	1	4.28 ± 0.16	1.81 ± 0.06	3.66 ± 0.17	0.49 ± 0.03	7.62 ± 0.42	0.14 ± 0.01	0.71 ± 0.09
	9	4.44 ± 0.19	1.78 ± 0.17	3.72 ± 0.14	0.62 ± 0.05*	6.25 ± 0.57*	0.17 ± 0.02*	0.83 ± 0.09
ND	—	4.28 ± 0.14	1.82 ± 0.18	3.67 ± 0.16	0.59 ± 0.04	6.44 ± 0.42	0.16 ± 0.01	0.61 ± 0.04
ND/EtSF-S/P	1	4.31 ± 0.17	1.93 ± 0.19	3.52 ± 0.14	0.78 ± 0.04**	4.62 ± 0.20**	0.22 ± 0.01**	0.78 ± 0.06*
	9	4.67 ± 0.15	2.09 ± 0.11	4.09 ± 0.18	0.74 ± 0.03**	5.52 ± 0.12*	0.18 ± 0.004	0.58 ± 0.05
HCLD-fed mice								
ND	—	4.15 ± 0.16	1.79 ± 0.10	3.70 ± 0.19	0.56 ± 0.04	6.71 ± 0.33	0.15 ± 0.01	0.45 ± 0.05
HCLD	—	5.59 ± 0.27**	1.24 ± 0.06**	3.60 ± 0.17	2.21 ± 0.12**	1.64 ± 0.06**	0.62 ± 0.02**	1.99 ± 0.15**
HCLD/EtSF-S	1	5.74 ± 0.23	0.97 ± 0.05^††^	3.57 ± 0.09	2.38 ± 0.13	1.52 ± 0.06	0.67 ± 0.03	2.18 ± 0.17
	3	5.68 ± 0.14	1.08 ± 0.05	3.70 ± 0.08	2.23 ± 0.08	1.67 ± 0.05	0.60 ± 0.02	1.98 ± 0.10
	9	5.68 ± 0.16	0.96 ± 0.07^††^	3.68 ± 0.09	2.31 ± 0.13	1.63 ± 0.08	0.63 ± 0.03	2.00 ± 0.12
HCLD/FF	0.1	3.95 ± 0.22^††^	0.77 ± 0.04^††^	3.34 ± 0.23	0.76 ± 0.07^††^	4.62 ± 0.31^††^	0.23 ± 0.02^††^	0.61 ± 0.06^††^
ND	—	4.23 ± 0.25	1.81 ± 0.05	3.64 ± 0.18	0.47 ± 0.03	7.90 ± 0.38	0.13 ± 0.01	0.71 ± 0.12
HCLD	—	5.06 ± 0.23*	1.01 ± 0.06**	3.22 ± 0.11	1.55 ± 0.05**	2.05 ± 0.11**	0.50 ± 0.02**	1.95 ± 0.15**
HCLD/EtSF-P	1	4.98 ± 0.15	1.04 ± 0.08	3.23 ± 0.09	1.61 ± 0.07	2.03 ± 0.08	0.50 ± 0.02	1.75 ± 0.13
	3	5.61 ± 0.20	0.92 ± 0.08	3.60 ± 0.14	1.74 ± 0.09	2.03 ± 0.09	0.50 ± 0.02	2.01 ± 0.10
	9	6.38 ± 0.20^††^	0.76 ± 0.06^††^	3.70 ± 0.14^†^	2.28 ± 0.09^††^	1.59 ± 0.07^††^	0.64 ± 0.03^††^	2.69 ± 0.13^††^
ND	—	4.28 ± 0.14	1.82 ± 0.18	3.67 ± 0.16	0.59 ± 0.04	6.44 ± 0.42	0.16 ± 0.01	0.61 ± 0.04
HCLD	—	5.10 ± 0.18**	0.73 ± 0.06**	3.74 ± 0.13	1.97 ± 0.11**	1.95 ± 0.12**	0.53 ± 0.04**	1.35 ± 0.13**
HCLD/EtSF-S/P	1	6.40 ± 0.16^††^	0.69 ± 0.07	4.26 ± 0.09^††^	2.88 ± 0.11^††^	1.50 ± 0.07^††^	0.68 ± 0.03^††^	2.14 ± 0.12^††^
	3	6.34 ± 0.31^††^	0.57 ± 0.04^†^	4.01 ± 0.19	2.80 ± 0.16^††^	1.45 ± 0.07^††^	0.70 ± 0.03^††^	2.33 ± 0.18^††^
	9	7.32 ± 0.23^††^	0.60 ± 0.05	4.38 ± 0.12^††^	3.41 ± 0.16^††^	1.30 ± 0.06^††^	0.78 ± 0.04^††^	2.94 ± 0.20^††^

Mice were fed with normal diet (ND) or hypercholesterolemic diet (HCLD) without and with the ethanolic extract of Schisandrae Fructus (SF) pulp, seed, or their combination (namely, EtSF-P, EtSF-S, and EtSF-P/S, resp.) and fenofibrate (FF) at the indicated doses (%, w/w), which was estimated on the basis of crude herbal material, for 10 days. Then serum total cholesterol (TC), triglyceride (TG), high-density lipoprotein (HDL), low-density lipoprotein (LDL), and non-HDL (N-HDL) levels, as well as HDL/LDL and LDL/HDL ratios, were measured. HCLD was constituted of 1% cholesterol and 0.3% bile salt (w/w). Values given are the means ± SEM, with *n* = 10. *P < 0.05, **P < 0.01 versus ND; ^†^ *P* < 0.05, ^††^ *P* < 0.01 versus HCLD. Statistical significant differences were determined using a one-way ANOVA followed by Dunnett's multiple comparisons test or post hoc analysis.

**Table 2:** Effects of EtSF supplementation on body weight and food/drug intake in normal and HCL mice.

Groups	Drug concentration (%, w/w)	Body weight (g) in D 1	Body weight (g) in D 10	Food intake (g/kg/day)	Drug intake (g/kg/day)
ND-fed mice					
ND	—	18.55 ± 0.09	28.70 ± 0.53	148.54	—
ND/EtSF-S	1	18.58 ± 0.09	28.10 ± 0.45	147.80	1.48
	9	18.56 ± 0.09	28.24 ± 0.53	151.48	13.63
ND/FF	0.1	18.52 ± 0.11	26.36 ± 0.65*	145.27	0.15
ND	—	18.14 ± 0.18	28.02 ± 0.45	158.60	—
ND/EtSF-P	1	18.08 ± 0.18	26.55 ± 0.52*	147.39	1.47
	9	18.03 ± 0.19	26.38 ± 0.41*	165.29	14.88
ND		18.52 ± 0.10	28.02 ± 0.51	154.29	—
ND/EtSF-S/P	1	18.48 ± 0.11	27.87 ± 0.50	146.88	1.47
	9	18.55 ± 0.11	28.42 ± 0.45	145.30	13.08
HCLD-fed mice					
ND	—	18.60 ± 0.16	28.48 ± 0.50	156.21	—
HCLD	—	18.68 ± 0.18	28.48 ± 0.49	143.35	—
HCLD/EtSF-S	1	18.55 ± 0.17	29.49 ± 0.53	145.75	1.46
	3	18.65 ± 0.16	28.48 ± 0.72	141.09	4.23
	9	18.62 ± 0.17	28.07 ± 0.47	141.31	12.72
HCLD/FF	0.1	18.67 ± 0.19	23.81 ± 0.62^††^	132.00	0.13
ND	—	18.14 ± 0.18	28.02 ± 0.45	158.60	—
HCLD	—	18.63 ± 0.22	27.74 ± 0.59	159.60	—
HCLD/EtSF-P	1	18.92 ± 0.34	28.17 ± 0.62	164.80	1.65
	3	18.45 ± 0.25	28.55 ± 0.79	182.16	5.47
	9	18.41 ± 0.29	27.06 ± 0.45	171.46	15.43
ND	—	18.52 ± 0.10	28.02 ± 0.51	154.29	—
HCLD	—	19.00 ± 0.26	27.37 ± 0.48	157.86	—
HCLD/EtSF-S/P	1	18.88 ± 0.24	29.04 ± 0.92	167.58	1.68
	3	19.14 ± 0.30	28.17 ± 0.47	172.16	5.17
	9	18.90 ± 0.30	27.47 ± 0.42	166.41	14.98

Experimental details were described in Table 1. The dosages (g/kg/day) based on crude herbal material were determined with the amount of ingested diet (g/kg/day) and drug concentrations in the diet. Values given are the means ± SEM, with *n* = 10. *P < 0.05 versus mice fed with ND; ^††^ *P* < 0.01 versus mice fed with HCLD. Statistical significant differences were determined using a one-way ANOVA followed by Dunnett's multiple comparisons test or post hoc analysis.

**Table 3:** A summary of results from the study.

	EtSF-S dietary supplement	EtSF-P dietary supplement	EtSF-S/P dietary supplement	FF dietary supplement
*ND-fed mice *				
Serum TC	—	—	—	↓
TG	—	—	—	↓
HDL	—	—	—	↓
LDL	—	↑	↑	—
ALT activity	—	—	—	↑
Hepatic TC	↓	—	↓	↓
TG	↓	↓	↓	↓
Glucose	↓	↓	↓	↓
Index	—	—	↑	↑
Biliary TC	—	—	—	↓
Fecal TC	—	↑	—	—
Body weight gain	—	↓	—	↓
*HCLD-fed mice* (*change versus ND-fed mice*)				
Serum TC (↑)	—	↑	↑	↓
TG (↓)	↓	↓	↓	↓
HDL (—)	—	↑	↑	—
LDL (↑)	—	↑	↑	↓
ALT activity (↑)	—	↓	↓	↑
Hepatic TC (↑)	↓	↓	↓	↓
TG (↑)	↓	↓	↓	↓
Glucose (↑)	↓	↓	↓	↓
Index (↑)	—	—	—	↑
Biliary TC (↑)	↑	—	↑(1%) ↓(9%)	↑
Fecal TC (↑)	—	—	↑	—
Body weight gain (—)	—	—	—	↓

↑: increased or elevated; ↓: decreased or inhibited; —: unaltered.

TC: total cholesterol; TG: triglyceride; HDL: high-density lipoprotein; LDL: low-density lipoprotein; ALT: alanine aminotransferase; FF: fenofibrate.
